# ACLY and CKD: A Mendelian Randomization Analysis

**DOI:** 10.1016/j.ekir.2022.04.013

**Published:** 2022-04-22

**Authors:** Pedrum Mohammadi-Shemirani, Michael Chong, Nicolas Perrot, Marie Pigeyre, Gregory R. Steinberg, Guillaume Paré, Joan C. Krepinsky, Matthew B. Lanktree

**Affiliations:** 1 Department of Biomarkers and Genetics, Population Health Research Institute, Hamilton, Ontario, Canada; 2 Experimental Program, Thrombosis and Atherosclerosis Research Institute, Hamilton, Ontario, Canada; 3 Department of Medical Sciences, McMaster University, Hamilton, Ontario, Canada; 4 Division of Endocrinology and Metabolism, Department of Medicine, McMaster University, Hamilton, Ontario, Canada; 5 Centre for Metabolism, Obesity, and Diabetes Research, McMaster University, Hamilton, Ontario, Canada; 6 Department of Biochemistry and Biomedical Sciences, McMaster University, Hamilton, Ontario, Canada; 7 Department of Pathology and Molecular Medicine, McMaster University, Hamilton, Ontario, Canada; 8 Department of Health Research Methods, Evidence & Impact, McMaster University, Hamilton, Ontario, Canada; 9 Division of Nephrology, Department of Medicine, McMaster University, Hamilton, Ontario, Canada; 10 Division of Nephrology, St. Joseph’s Healthcare Hamilton, Hamilton, Ontario, Canada

**Keywords:** chronic kidney disease, citrate, fibrosis, genomics, Mendelian randomization

## Abstract

**Introduction:**

Adenosine triphosphate-citrate lyase (ACLY) inhibition is a therapeutic strategy under investigation for atherosclerotic cardiovascular disease, nonalcoholic steatohepatitis, and metabolic syndrome. Mouse models suggest that ACLY inhibition could reduce inflammation and kidney fibrosis. Genetic analysis of ACLY in chronic kidney disease (CKD) has not been performed.

**Methods:**

We constructed a genetic instrument by selecting variants associated with *ACLY* expression in the expression quantitative trait loci genetics consortium (eQTLGen) from blood samples from 31,684 participants. In a 2-sample Mendelian randomization analysis, we evaluated the effect of genetically predicted *ACLY* expression on the risk of CKD, estimated glomerular filtration rate (eGFR), and albumin-to-creatinine ratio (ACR) using the CKD Genetics (CKDGen) consortium, UK Biobank, and the Finnish Genetics (FinnGen) consortium totaling 66,396 CKD cases and 958,517 controls.

**Results:**

*ACLY* is constitutively expressed in all cell types including in whole blood. The genetic instrument included 13 variants and explained 1.5% of the variation in whole blood *ACLY* gene expression. A 34% reduction in *ACLY* expression score was associated with a 0.04 mmol/l reduced low-density lipoprotein (LDL) cholesterol (*P* = 3.4 × 10^−4^) and a 9% reduced risk of CKD (stages 3, 4, 5, dialysis, or eGFR < 60 ml/min per 1.73 m^2^) (odds ratio [OR] = 0.91, 95% CI: 0.85–0.98, *P* = 0.008), but no association was observed with either eGFR or ACR.

**Conclusion:**

Mendelian randomization analyses revealed that genetically reduced ACLY expression was associated with reduced risk of CKD but had no effect on either eGFR or ACR. Further evaluation of ACLY in kidney disease is warranted.

## Graphical abstract

**Figure:**
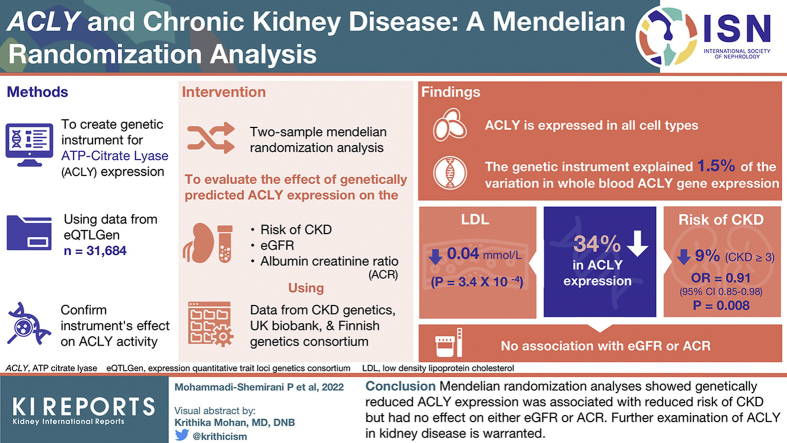

CKD remains an underappreciated cause of morbidity and mortality that is increasing in prevalence. CKD is a complex multifactorial syndrome and the development of successful therapies has been challenging. Despite success with renin-angiotensin-aldosterone system blockade and sodium-glucose cotransporter 2 inhibitors, there remains a significant need for the development of novel therapeutic targets for CKD. Metabolic dysregulation, inflammation, and fibrosis are of great interest as they are key features of CKD that are not directly targeted by current treatments.

ACLY is a cytosolic enzyme that catalyzes the conversion of citrate into acetyl coenzyme A, making it a critical intracellular switch to divert citrate between either oxidative phosphorylation in the citric acid cycle or to increase intracellular acetyl coenzyme A.^1^^,^^2^ Cytosolic acetyl coenzyme A can lead to either fatty acid and cholesterol synthesis or be shifted into the nucleus where it modifies histone hyperacetylation at the H3K9/14 and H3K18 genomic marks promoting transcription of inflammatory and fibrotic pathways, such as nuclear factor kappa B and transforming growth factor β.^3^ In both mice and humans, ACLY inhibition in the liver effectively reduces LDL cholesterol synthesis^1^^,^^2^; thus, ACLY inhibition was first evaluated as a treatment for dyslipidemia and cardiovascular disease. In a Mendelian randomization study of ACLY and cardiovascular disease, Ference *et al.*^4^ reported an 18% reduction in cardiovascular events (OR = 0.82, 95% CI: 0.78–0.87, *P* = 4.0 × 10^−14^) per 10 mg deciliter LDL cholesterol level reduction by *ACLY* genetic score. Pharmacologically, bempedoic acid is a small molecule ACLY inhibitor that safely lowered LDL cholesterol in multiple human clinical trials and a large cardiovascular outcome trial is ongoing.^5^ Lowering LDL cholesterol using statin medications has not proven effective for slowing the progression of CKD in randomized controlled trials^6^ and genetically increased LDL cholesterol is not associated with CKD in Mendelian randomization studies.^7^ However, findings from preclinical mouse models suggest that ACLY inhibition could reduce kidney fibrosis and inflammation making it an intriguing therapeutic target for CKD.^8^^,^^9^ Insulin resistance, obesity, nonalcoholic fatty liver disease, and proinflammatory cytokines, such as interleukin-4 and interleukin-2, all of which are risk factors for CKD progression, lead to further increases in ACLY activity.^1^^,^^10^^,^^11^

Mendelian randomization analysis is a valuable tool, rapidly growing in popularity, for using genetics to evaluate whether risk factors are causal contributors.^12^ Owing to Mendel’s law of independent assortment, your genotype at one location in the genome is independent of your genotype throughout the rest of the genome. Variants that are located close to each other on a chromosome are the exception and are more likely to be inherited together, a correlation called linkage disequilibrium. In Mendelian randomization, genetic variants that alter a risk factor are used for a “natural” randomized trial to evaluate the effect of the risk factor on an outcome, reducing unmeasured or residual confounding between the risk factor and the outcome. A single genetic variant often fails to explain enough variation in the risk factor to evaluate the risk factor’s role in a disease, so independent genetic variants (i.e., not in linkage disequilibrium) are combined into scores at the individual level, or the effects of numerous independent variants are jointly assessed using regression.^13^ The genetic variants selected for inclusion in a Mendelian randomization analysis are referred to as the genetic instrument. Underlying assumptions of Mendelian randomization analysis need to be addressed with sensitivity analyses including the following: (i) the instrument explains a significant proportion of variation in the exposure of interest, (ii) no association exists between the instrument and confounding factors, and (iii) no association exists between the instrument and alternative causal pathways to the outcome except by the exposure.^13^

Quantitative trait loci (QTL) are genetic variants associated with continuous molecular traits, such as gene expression (eQTL), DNA methylation (meQTL), or protein concentration (pQTL).^14^ The eQTLgen performed a meta-analysis of genome-wide association studies of each genes’ expression to identify eQTL,^15^ creating a valuable resource for selecting genetic variants to be included in genetic instruments for Mendelian randomization analyses.^14^ In the current study, as opposed to evaluating a traditional risk factor or biomarker, we performed a Mendelian randomization study of *cis* (i.e., nearby) genetic variants that explain a proportion of the variation in *ACLY* expression.

Analyses of human genetic data regarding the importance of ACLY in kidney disease have yet to be reported. Using data from eQTLGen, we sought to create a genetic instrument for *ACLY* expression, evaluate the association between the instrument and LDL cholesterol to confirm the instruments effect on ACLY activity, and then assess the effect of the ACLY instrument on kidney phenotypes, including prevalent CKD, eGFR, and ACR in population-level biobank data.

## Methods

### Creation of a Genetic Instrument for Human ACLY Expression

We first evaluated the tissue-specific expression profile of *ACLY* in humans in the Genotype-Tissue Expression Portal.^16^ We identified genetic variants associated with *ACLY* expression in the eQTLGen Consortium data set (https://www.eqtlgen.org/). eQTLGen performed genome-wide association studies assessing the association between more than 11 million variants and the expression level of 16,989 genes in 31,684 participants from 37 cohorts and 3 gene expression platforms to identify variants associated with whole blood gene expression (i.e., eQTLs).^15^ We selected the noncoding *cis* genetic variants within 500 kilobases of the *ACLY* gene that were associated with *ACLY* expression in the whole blood (*P* < 0.05). Identified variants were then compared with the UK Biobank genotype data set to avoid inclusion of eQTLGen variants that were unavailable in UK Biobank data set. A prune-and-thresholding approach was used to identify independent genetic variants associated with *ACLY* expression. After identifying the most strongly associated variant, genetic variants in linkage disequilibrium with an r^2^ > 0.01 in 1000 genome participants of European ancestry were pruned and discarded using the *clump* command in PLINK (http://zzz.bwh.harvard.edu/plink/).

### Study Outcomes

eGFR was calculated from serum creatinine (eGFR_Crea_) or cystatin (eGFR_Cys_), age, and sex using the CKD-EPI equation.^17^ The primary outcome was the presence of CKD stage 3 or worse, defined as prevalent eGFR_Crea_ or eGFR_Cys_ < 60 ml/min per 1.73 m^2^. Secondary kidney outcomes included log-transformed quantitative eGFR and urinary ACR. Urinary albumin was determined in a spot urine sample in UK Biobank; those with no detectable urinary albumin were set to the lower limit of detection and the ACR was log-transformed. We evaluated LDL cholesterol and the correlated apolipoprotein B as positive controls for our *ACLY* expression instrument. In further exploratory sensitivity analyses, we evaluated the previously defined and reported “rapid3” (>3 ml/min per 1.73 m^2^ per yr) and “CKDi25” (25% or more decline in eGFRcrea or eGFRcys and crossing >60 ml/min per 1.73 m^2^) phenotypes.^18^

### Study Populations

After the genetic variants were selected, we first evaluated the strength of the *ACLY* expression genetic instrument in eQTLGen based on the exposure r^2^ and F statistic using the 2-sample MR package (https://rdrr.io/github/MRCIEU/TwoSampleMR/). Second, we assessed the cumulative effect of the genetic variants associated with *ACLY* expression by calculating an individual-level effect-weighted genetic score and testing its association with both LDL cholesterol and kidney traits in the European ancestry participants of the UK Biobank (n = 343,648). Third, we evaluated the association in the 2019 Wuttke *et al.*^19^ public summary-level CKD association results of CKDGen (n = 522,093) accessed by https://ckdgen.imbi.uni-freiburg.de/. Finally, data from the FinnGen consortium release 5 (n = 178,274) were accessed by www.finngen.fi. Data analyses in UK Biobank were performed under UK Biobank application number 15255, whereas FinnGen and CKDGen provide publicly available data. Notably, there is no sample overlap between these 3 population-level data sets. The UK Biobank has approval from the North West Multi-Center Research Ethics Committee. All remaining analyses were based on publicly available summary statistics; therefore, no individual consent nor ethical approval from an institutional review board was necessary.

We also evaluated the effect of the *ACLY* expression instrument on eGFR in the summary-level results of the Stanzick *et al.*^20^ eGFR meta-analysis, which includes the data from both CKDGen and UK Biobank (n = 1,201,930) and on the “rapid3” (34,874 cases and 107,090 controls) and “CKDi25” (19,901 cases and 175,244) control phenotypes as defined in the meta-analysis of Gorski *et al.*^18^ Note that these additional analyses include the same overlapping CKDGen and UK Biobank samples as the primary analysis and should not be viewed as independent tests nor replications of primary analyses.

### Evaluation of Rare Genetics Variants in ACLY by Population Sequencing

We next evaluated the prevalence of *ACLY* loss-of-function variants in the general population using the loss-of-function transcript effect estimator in gnomAD (https://gnomad.broadinstitute.org/).^21^ Genes that are essential for life contain less genetic variation in the general population.^21^ Although the presence or absence of constraint against loss-of-function variants cannot nominate or exclude a gene as a drug target,^22^ they can be helpful to estimate the prevalence of those with haploinsufficiency. Second, we looked for a lack of rare variants in those with coded “N18 Chronic Renal Failure” (n = 9856) or “N17 Acute Renal Failure” (n = 5015) of 281,585 UK Biobank participants in the gene-based association summary statistics phenome-wide association results database Genebass (https://genebass.org/), and finally we tested for association of rare *ACLY* variants with lipid and kidney phenotypes in 173,688 UK Biobank participants with available exome and quantitative phenotype data. A logistic regression model was used to test the association between *ACLY* rare variant carrier status and each outcome adjusted for age, age^2^, sex, assessment center, and the first 20 genetic principal components of ancestry.

### Statistical Analysis

Where individual-level genotype data were available (i.e., in the UK Biobank where every individual’s genotype is available), we constructed an *ACLY* eQTL genetic instrument for each individual. For each variant in the instrument, the number of expression-increasing alleles was coded as 0, 1, and 2 and multiplied by its normalized effect on *ACLY* expression. The specific genetic variants included in the genetic instrument and their weights are provided in Supplementary Table S1. We then performed regression to evaluate the relationship between the *ACLY* instrument and kidney traits, including adjustment for age, sex, and 20 principal components of ancestry.

Where only summary-level association results were available (i.e., the effect of each variant on the trait of interest in the complete sample), we used the inverse variance-weighted method regressing the genetic effect estimate of each variant on *ACLY* expression against the genetic effect estimate of each variant on the kidney trait. The weighted median estimator was also calculated as it is consistent even when up to 50% of the information comes from invalid instruments.^23^ The effect estimates from the UK Biobank and summary-level CKDGen and FinnGen cohorts were combined across studies in a fixed-effect inverse variance-weighted meta-analysis.

To evaluate the possibility of unmeasured directional pleiotropy, we also performed Egger Mendelian randomization. As the effect of a variant on *ACLY* expression goes down to zero, its effect on the kidney trait should in theory also go to zero. Egger Mendelian randomization analysis allows for a non-zero Y-intercept in the Mendelian randomization regression line. Should a bias exist, the regression line does not go through the origin, and an effect on kidney traits seems to remain non-zero even as the effect of the variants on *ACLY* expression approaches zero. The Egger Mendelian randomization intercept test evaluates whether the intercept is significantly different than zero. The primary limitation of Egger Mendelian randomization is a decrease in power; thus, it is provided as a sensitivity analysis to evaluate for the possible presence of directional pleiotropy. In an additional sensitivity analysis, we used Mendelian Randomization Pleiotropy RESidual Sum and Outlier (MRPRESSO) to detect the presence of horizontal pleiotropy and remove outlier variants. All analyses were conducted in accordance with STROBE-MR: guidelines for strengthening the reporting of Mendelian randomization studies (see STROBE-MR checklist in Supplementary Materials). All statistical analyses were conducted using R version 3.3.2 software.

## Results

### Development of the ACLY Expression Genetic Instrument

**Figure 1:**
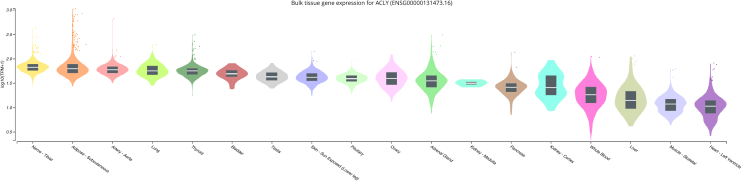
*ACLY* is ubiquitously expressed including in the whole blood. ACLY, adenosine triphosphate-citrate lyase; EBV, Epstein-Barr virus; TPM, transcripts per million. Data source: GTEx Analysis Release V8 (dbGaP Accession phs000424.v8.p2).

Before developing a genetic instrument for *ACLY* expression, we verified that *ACLY* is ubiquitously expressed with similar expression levels (transcripts per million reads) in the kidney, liver, and whole blood (Figure 1). A total of 13 noncoding variants within 500 kilobases of *ACLY* were identified as independently associated with the quantity of *ACLY* expression (*P* < 0.05) and included in the genetic instrument (Supplementary Table S1). Cumulatively, the instrument explained 1.5% of whole blood expression of *ACLY* (*F* = 32.9), which exceeds the common requirement for Mendelian randomization instruments to be greater than 10.^24^ In the UK Biobank, the *ACLY* expression genetic instrument was associated with the positive control LDL cholesterol (β = 0.04 mmol/l decrease per 34% relative reduction in *ACLY* eQTL genetic instrument , *P* = 3.4 × 10^−4^) and apolipoprotein B (β = 0.01 mmol/l decrease per 34% relative reduction in *ACLY* eQTL genetic instrument, *P* = 2.7 × 10^−5^) (Supplementary Table S2). These observations are consistent with the *ACLY* eQTL genetic instrument affecting *ACLY* activity.

### Association of ACLY Expression Genetic Instrument With Kidney Outcomes

**Figure 2:**
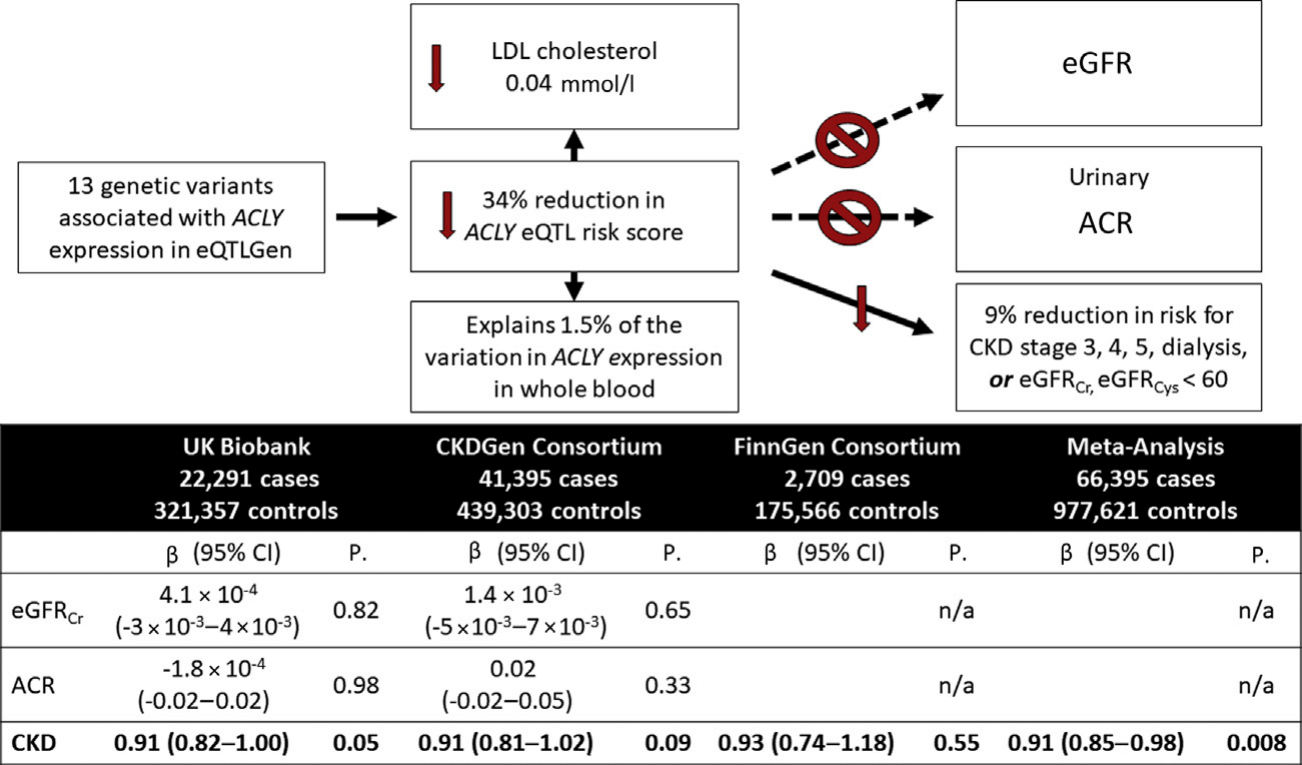
Mendelian randomization analysis supports that reduced *ACLY* expression creates reduced risk of CKD without affecting eGFR or ACR. β given as % change in trait or odds ratio per 34% reduction in genetically determined *ACLY* eQTL genetic instrument. eGFR and ACR were log-transformed. ACLY, adenosine triphosphate-citrate lyase 2; ACR, albumin-to-creatinine ratio; CKD, chronic kidney disease; eGFR, estimated glomerular filtration rate; eGFR_cr_, eGFR was calculated from serum creatinine; eGFR_cys_, eGFR was calculated from serum cystatin; eQTL, expression quantitative trait loci; eQTLGen, eQTL genetics consortium; n/a, not applicable.

Meta-analysis of the primary CKD phenotype across UK Biobank, CKDGen, and FinnGen data including 66,396 CKD cases and 958,517 controls revealed a significant association between reduced *ACLY* expression and reduced risk of CKD (OR = 0.91, 95% CI: 0.85–0.98 per 34% relative reduction in *ACLY* eQTL genetic instrument; *P* = 0.008; Figure 2 and Supplementary Figure S1). The effect estimates were concordant in the 3 contributing studies: UK Biobank, CKDGen, and FinnGen. The UK Biobank White British ancestry participants included 22,291 cases with prevalent CKD stage 3, 4, or 5, or eGFR_Crea_ or eGFR_Cys_ of <60 ml/min per 1.73 m^2^, and 321,357 participants without prevalent CKD, and we observed the same effect as the meta-analysis (OR = 0.91 per 34% relative reduction in *ACLY* eQTL genetic instrument, 95% CI: 0.82–1.00, *P =* 0.05). The *ACLY* eQTL instrument-CKD association was not statistically significant in 2-sample Mendelian randomization analysis of the summary level results of either CKDGen or FinnGen, but the effect estimates were very similar to meta-analysis (OR = 0.91 per 34% relative reduction in *ACLY* eQTL genetic instrument, 95% CI: 0.81–1.02, *P =* 0.09 in CKDGen, including 41,395 cases and 439,303 controls [Supplementary Figure S1], and OR = 0.93, 95% CI: 0.74–1.18, *P* = 0.55 in FinnGen, including 2709 cases and 175,566 controls [Supplementary Figure S2]). The Egger regression found no significant evidence for a nonzero intercept and MRPRESSO did not identify evidence of horizontal pleiotropy or outlier variants.

In both individual-level Mendelian randomization analysis within the UK Biobank White British ancestry participants and summary-level Mendelian randomization analysis with CKDGen, our analysis displayed no association between the *ACLY* eQTL genetic instrument and any of eGFR_Crea_, eGFR_Cys_, or ACR (*P* > 0.3). An eGFR meta-analysis published by Stanzick *et al.*^20^ included both of CKDGen and UK Biobank populations representing the same participants as in our primary analysis*.* We performed 2-sample Mendelian randomization of their eGFR meta-analysis summary results as a sanity check and obtained similar results to our primary analysis (eGFRcrea *P* = 0.83 and eGFRcys *P* = 0.73; Supplementary Table S3).^20^ Concordant with the CKD observation, Mendelian randomization revealed significant association between reduced *ACLY* expression and reduced risk of the dichotomous “Rapid3” phenotype (OR = 0.88, 95% CI: 0.78–1.00, *P* = 0.05) with the weighted median model, whereas the inverse variance weighted model revealed similar effect size albeit above the significance threshold (OR = 0.88, 95% CI: 0.76–1.01, *P* = 0.07). There was no association found with the dichotomous “CKDi25” phenotype (*P* > 0.5).^18^

### Population-Level Analysis of Rare Genetic Variants in ACLY

No monogenic phenotype has been reported from rare pathogenic variants in *ACLY.* Exome sequencing of control populations reported in gnomAD identified 15 loss-of-function *ACLY* genetic variants and 0 homozygous human *ACLY* “knockouts” among 141,456 sequenced exomes. This corresponds to a carrier prevalence of heterozygous *ACLY* loss-of-function mutations of 1 in 1221 people in the general population*.* There were 64% fewer missense variants (95% CI: 59%–70%) and 24% fewer loss-of-function mutations (95% CI: 16%–38%) compared with the expectation based on the distribution of rare variants in all genes in the genome based on the gnomAD *observed/expected score*. In the 281,585 UK Biobank participants included in the Genebass resource, there was no lack of loss-of-function variants in participants with “N18 Chronic Renal Failure” (*P* = 0.11) or “N17 Acute Renal Failure” (*P* = 0.63), nor with any of the other 1756 phenotypes in the GeneBass phenome-wide association results below a Bonferroni-corrected significance (*P* < 5 × 10^−5^). In the 173,688 participants of UK Biobank with individual-level exome and quantitative trait data available to our group, there were 51 carriers with heterozygous-predicted loss-of-function variants and there was no association with the positive control, LDL cholesterol (*P* = 0.91) (Supplementary Tables S4–S6).

## Discussion

Using Mendelian randomization analysis of *cis* genetic variants associated with *ACLY* expression and population-scale genetic studies of kidney phenotypes, we found evidence that genetically reduced *ACLY* expression was associated with reduced risk of CKD. There was also a trend toward association of reduced *ACLY* expression and avoidance of the “rapid3” >3 ml/min per 1.73 m^2^ loss per year phenotype. However, we found no association between genetically predicted *ACLY* expression and either eGFR or urinary ACR. We also found no association between rare mutations in *ACLY* and either our positive control, LDL cholesterol, nor any kidney phenotype. Although we are unable to make a firm conclusion regarding a causal role for ACLY in kidney disease, our results suggest that further investigation is warranted.

The discordant association of the *ACLY* eQTL genetic instrument with CKD but not with reduced eGFR or increased ACR creates skepticism regarding the validity of the ACLY-CKD observation. Despite the consistency of the observed effect size across 3 large population studies, a false positive result remains a possibility. However, although counterintuitive, the genetic determinants of CKD could differ from the genetic determinants of eGFR and ACR. A genetic variant associated with higher eGFR in the general population could also increase the risk of CKD if it increases the likelihood of hyperfiltration in certain scenarios. Similarly, a genetic variant that promotes kidney fibrosis could increase the risk of CKD development without a measurable effect on eGFR or ACR, especially when evaluating populations at the time of largely normal kidney function. Similar complex genetic architectures, where differing genetic risk factors exist for continuous traits in the general population and discrete diagnostic thresholds at the extreme of the trait, have been observed for other traits. For example, even though type 2 diabetes can be diagnosed by elevated fasting glucose, genetic variants are variably associated with fasting glucose and risk of type 2 diabetes.^25^ CKDGen has been a monumental achievement furthering our understanding of the genetic determinants of eGFR, but further ascertainment of patients with advanced CKD (i.e., CKD stages 3, 4, and 5) is required to further evaluate the genetic architecture of CKD.

The largest clinical trial of pharmacologic inhibition of ACLY was the Cholesterol Lowering by bempedoic acid, an ACLY-Inhibiting Regimen (CLEAR) Harmony trial that enrolled 2230 people with high vascular risk and LDL cholesterol greater than 70 mg per deciliter despite maximally tolerated statin therapy. Daily bempedoic acid for 52 weeks lowered mean LDL cholesterol levels by 0.50 mmol/l (18%) and did not increase the incidence of adverse events compared with placebo.^26^ We observed a 0.04 mmol/l reduction in LDL cholesterol per 34% reduction in *ACLY* eQTL genetic instrument, consistent with only 8% of the ACLY inhibition observed with bempedoic acid. Thus, although we suspect that mechanisms other than LDL reduction contribute to a protective effect of ACLY inhibition on CKD, as bempedoic acid has a much larger effect on ACLY inhibition than our *ACLY* eQTL genetic score, it is possible for bempedoic acid to have a larger effect on CKD prevention than the 9% reduction we observed with the *ACLY* eQTL genetic instrument. The CLEAR Harmony trial was of insufficient duration to evaluate cardiovascular outcomes, but we await the results of the CLEAR Outcomes trial that has finished recruitment of 14,000 statin-intolerant patients in 2019. Although not a prespecified end point of the CLEAR Outcomes trial, and likely a paucity of kidney outcomes in the study, retrospective analysis of differences in the development of CKD between those treated with bempedoic acid or placebo would be of great interest.

It is unclear whether beneficial effects of ACLY inhibition against cardiovascular disease will be mediated by LDL cholesterol lowering or alternate pathways. The Ference *et al.*^27^ Mendelian randomization analysis of ACLY inhibition and major cardiovascular events was criticized as the variants in the *ACLY* genetic instrument were selected based on their association with LDL cholesterol and not ACLY activity, and the studied variants were only modestly associated with LDL cholesterol levels or coronary artery disease in additional populations. We used a superior approach by selecting variants associated with the quantity of *ACLY* expression (i.e., eQTL) and a more conservative linkage disequilibrium pruning threshold. As a result, none of the variants in the current study exactly overlap with those used in the study of Ference *et al.*^27^ of ACLY. Nonetheless, we still found a strong association of our ACLY eQTL genetic instrument and LDL cholesterol in the UK Biobank, which served as a positive control for this study.

An important difference between Mendelian randomization analysis of *ACLY* expression and clinical trials with bempedoic acid is related to the tissue-targeted effects of bempedoic acid. *ACLY* is ubiquitously expressed, and we would expect the *ACLY* eQTL genetic instrument to reflect changes in *ACLY* expression in all tissue types to some extent.^15^^,^^28^ In contrast, the selectivity of bempedoic acid for inhibiting ACLY is the requirement for conversion into bempedoic acid-CoA by long-chain acyl-CoA synthetase-1 (SLC27A2), which is minimally expressed in most cell types.^2^ However, Genotype-Tissue Expression Portal indicates that the kidney cortex is second only to the liver for *SLC27A2* expression suggesting bempedoic acid would also inhibit ACLY in the kidney (Supplementary Figure S3). As ACLY catalyzes the conversion of citrate into acetyl coenzyme A, it is a reasonable hypothesis that inhibition of ACLY would lead to an increase in intracellular citrate concentrations. Urinary citrate is inversely correlated with glomerular filtration rate and acid retention.^29^ Low urinary citrate has also been recognized as a risk factor in autosomal dominant polycystic kidney disease.^30^ Whether ACLY inhibition would alter urinary citrate concentrations or whether urinary citrate could be a biomarker of kidney ACLY inhibition is yet unknown.

Strengths of this work include using the eQTLGen consortium to select genetic variants associated with *ACLY* expression and large population-based cohorts for the 2-sample Mendelian randomization analysis. Mendelian randomization studies using eQTL, meQTL, and pQTL data are going to grow in popularity for evaluating putative therapeutic targets in kidney disease going forward. All Mendelian randomization analyses are limited by the possibility of unmeasured pleiotropy, where the outcome is affected by the genetic variant through a pathway different than the exposure, but the possibility of pleiotropy is greatly reduced using a 13-variant instrument nearby the encoding gene as opposed to hundreds of variants throughout the genome. The eQTLGen, CKDGen, and UK Biobank data available are primarily of White European ancestry, and improving the ancestral diversity of genomic studies is a recognized ethical and scientific imperative.^31^ We tested variants associated with *ACLY* expression in the blood, which is likely an imperfect proxy for *ACLY* expression and protein activity within the kidney tissues, but testing for association with LDL cholesterol concentration serves as a positive control for the effectiveness of our ACLY genetic instrument. Finally, the current study evaluated *ACLY* as a candidate due to biological plausibility and ongoing clinical trials of bempedoic acid. We anticipate that agnostic study designs that evaluate the eQTLs of all genes in the genome (called transcriptome-wide association studies [TWAS]) or the protein concentration QTLs of all genes in the genome (called proteo-genomic association studies) will become common.^32^, ^33^, ^34^

In conclusion, Mendelian randomization analyses found that genetically reduced *ACLY* expression is associated with reduced risk of CKD but had no effect on either eGFR or ACR. Further evaluation of ACLY in kidney disease, including evaluation of the effects of ACLY inhibition in currently ongoing clinical trials, is warranted.

## Disclosure

GP has received consulting fees from Bayer, Sanofi, Bristol-Myers Squibb, Lexicomp, and Amgen and support for research through his institution from Sanofi and Bayer. GRS is a cofounder and shareholder of Espervita Therapeutics. McMaster University has received funding from Espervita Therapeutics, Esperion Therapeutics, Novo Nordisk, and Poxel Pharmaceutical for research conducted in the laboratory of GRS. GRS has received consulting/speaking fees from AstraZeneca, Eli Lilly, Esperion Therapeutics, Merck, Poxel Pharmaceuticals, and Takeda. MBL has received speaker and advisory fees from Otsuka, Reata, Bayer, and Sanofi Genzyme. The funders played no role in the design, analysis, or interpretation of this work. All the other authors declared no competing interests.

## Supplementary Material

Supplementary File (PDF)

**Figure S1.** Two-sample Mendelian randomization plots of the instrument effect on *ACLY* expression and risk of CKD (a), urine ACR (b), and eGFR (c) in CKDGen.

**Figure S2.** Two-sample Mendelian randomization plots of the instrument effect on ACLY expression and risk of CKD in FinnGen.

**Figure S3.** GTEx tissue-specific expression of *SLC27A2* which is required for the activation of bempedoic acid.

**Table S1.** Markers included in *ACLY* expression genetic instrument from eQTLGen.

**Table S2.** Mendelian randomization of *ACLY* expression instrument with lipid and kidney traits in the UK Biobank.

**Table S3.** Two-sample summary-level Mendelian randomization analyses in CKDGen and FinnGen.

**Table S4.** Rare variant association results for UK Biobank.

**Table S5.** Predicted loss-of-function *ACLY* rare variants observed in UK Biobank participants.

**Table S6.** Predicted deleterious *ACLY* rare variants observed in UK Biobank participants.

**STROBE Statement.**
