# Supplementary material for: ACLY and CKD: A Mendelian Randomization Analysis
Source: Kidney Int Rep. 2022 Apr 22;7(7):1673–81. doi: 10.1016/j.ekir.2022.04.013 (PMC9263230; doi:10.1016/j.ekir.2022.04.013)
Supplement: Supplementary File (PDF) [file mmc1.pdf]

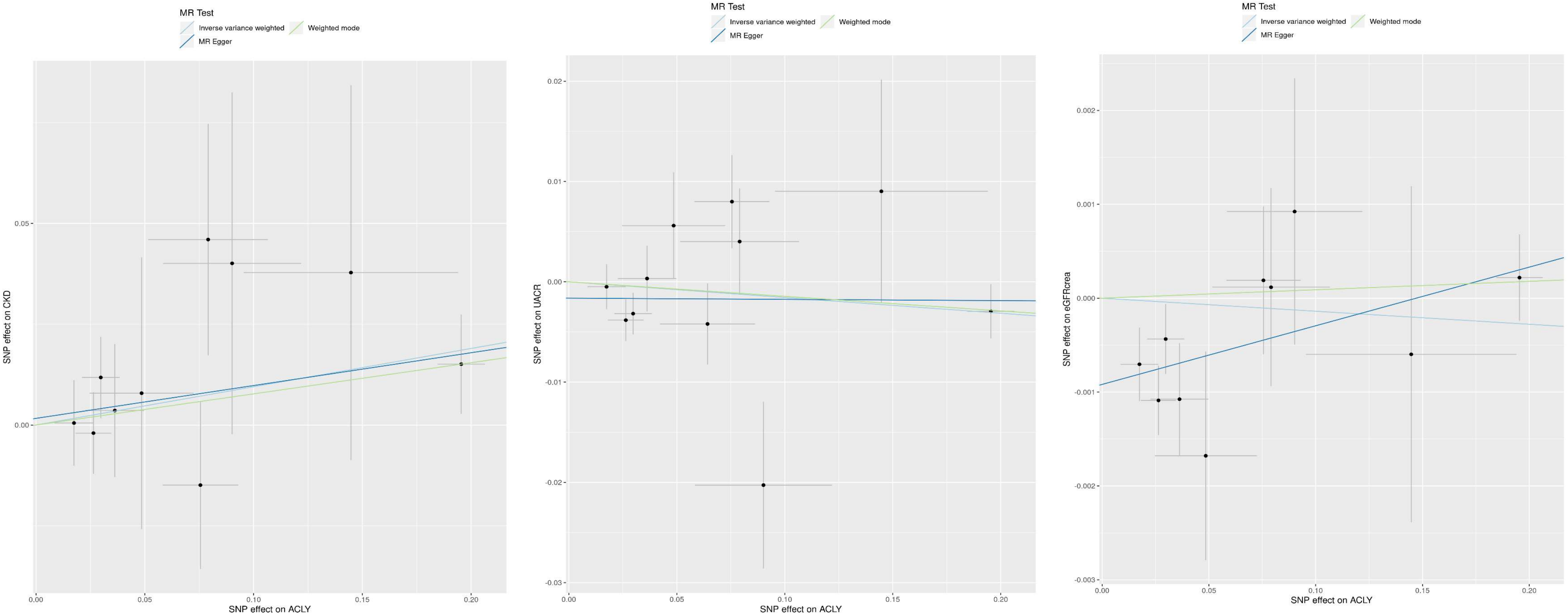

Figure S1. Two sample Mendelian randomization plots of the instrument effect on *ACLY* expression and risk of CKD (A), Urine ACR (B), and eGFR (C) in CKDGen.

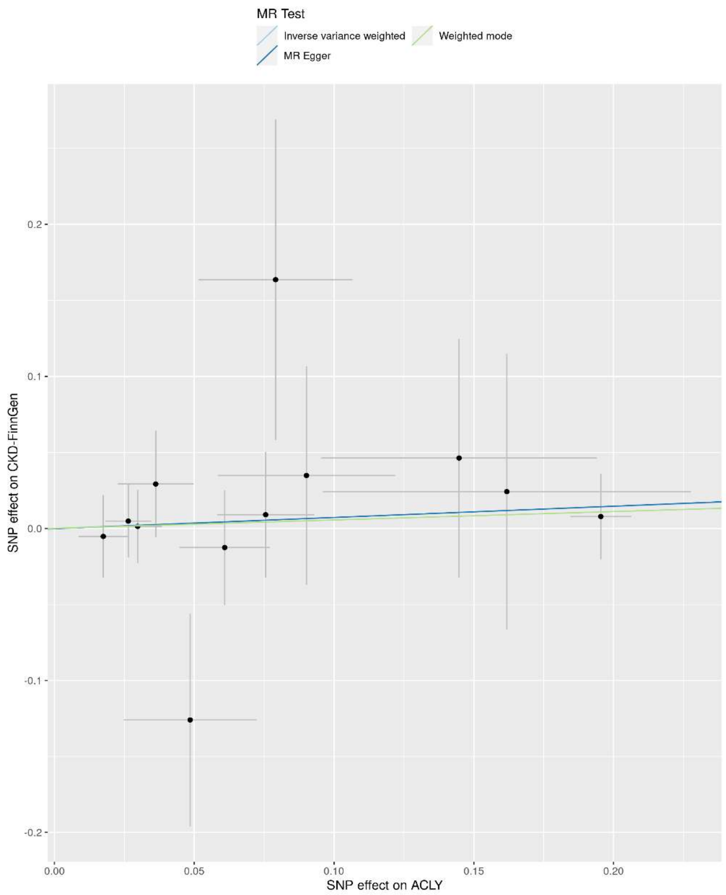

Figure S2. Two sample Mendelian randomization plots of the instrument effect on *ACLY* expression and risk of CKD in FinnGen.

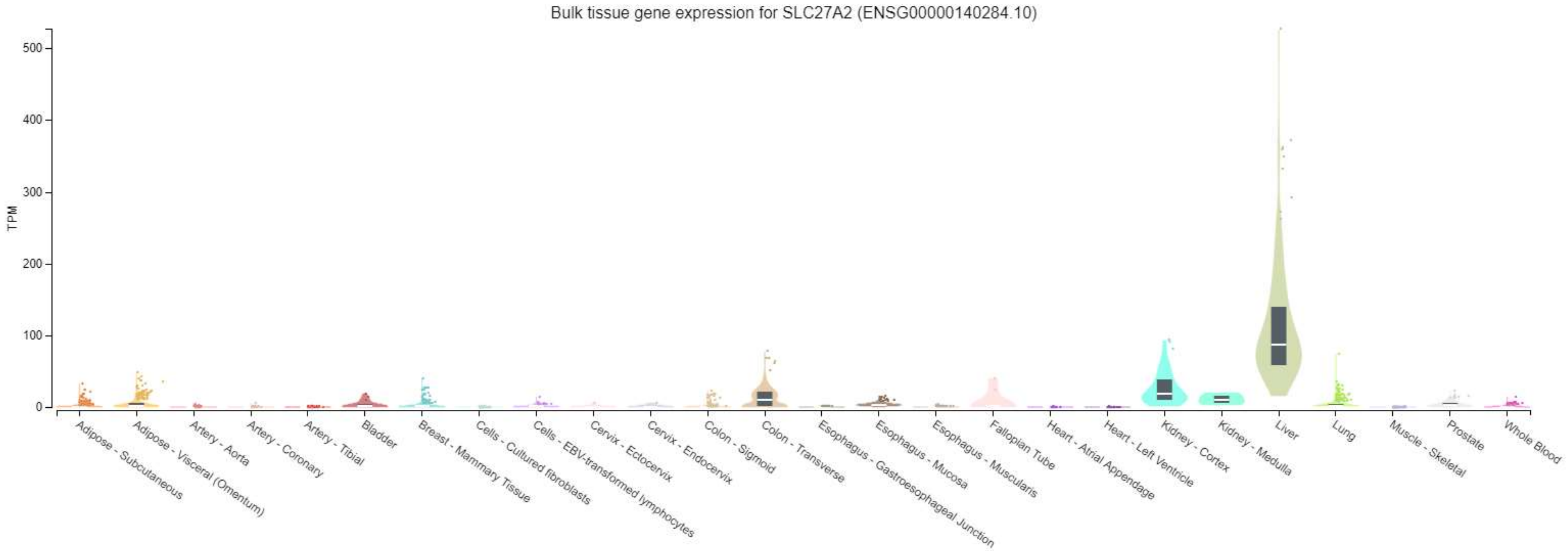

Figure S3. GTEx tissue-specific expression of *SLC27A2* which is required for the activation of bempedoic acid.

Table S1. Markers included in *ACLY* expression genetic instrument from eQTLGen

chr	SNP	position	Z-score	SE	effect_allele	other_allele	t Allele Frequ	*P*	n
17	rs34200091	40014216	0.20	0.011	G	A	0.16	3.70E-72	31529
17	rs79995639	40232810	0.076	0.017	C	T	0.94	1.34E-05	28095
17	rs35967904	40126187	0.061	0.016	A	G	0.90	0.00017	22137
17	rs4273091	39820170	0.030	0.0087	T	G	0.54	0.00062	26646
17	rs2354155	40546652	0.026	0.0082	G	A	0.52	0.0013	29653
17	rs148395774	40444507	0.14	0.049	C	T	0.01	0.0033	15551
17	rs149039453	39917954	0.064	0.022	A	G	0.95	0.0035	21347
17	rs117889290	40015853	0.079	0.028	C	T	0.97	0.0041	21851
17	rs142356140	40305556	0.090	0.032	T	C	0.98	0.0045	27158
17	rs80313087	39669071	0.036	0.014	A	G	0.11	0.0075	28433
17	rs138959129	40452429	0.16	0.066	C	A	0.01	0.014	7947
17	rs148029598	40000685	0.049	0.024	T	C	0.96	0.042	24814
17	rs1378496	39651572	0.017	0.009	G	A	0.28	0.049	31529

Effect size and P value taken from eQTLGenetics consortium

Table S2. Mendelian Randomization of *ACLY expression* instrument with lipid and kidney traits in the UK Biobank

UKBB	cases	controls	n	Beta*	SE	OR	Lower 95%CI	Upper 95%CI	*P*	P_het
Apolipoprotein B			325999	-0.012	0.0029		-0.018	-0.0065	2.69E-05	0.22
LDL cholesterol			326987	-0.038	0.011		-0.06	-0.017	0.00034	0.59
C reactive protein			326881	0.0024	0.053		-0.1	0.1	0.97	0.43
CKD	22291	321357		-0.098	0.05	0.91	0.82	1.00	0.05	0.38
eGFR_crea_CKDEpi			327416	0.00041	0.00185		-0.00322	0.00404	0.82	0.25
eGFR_cys_CKDEpi			327558	0.0005	0.00219		-0.00376	0.00485	0.8	0.47
Urine ACR			333804	-0.00018	0.0088		-0.017	0.018	0.98	0.2

CKD defined as CKD G3, G4, G5 or eGFRCrea or eGFRCys < 60 ml/min/1.73m2
* Beta represents:
ApoB: g/L per 1 SD change in eQTL score
LDL: mmol/L per 1 SD change in eQTL score
CRP: mg/L per 1 SD change in eQTL score
CKD: OR per 1SD change in eQTL score
eGFRCr: % change per 1SD change in eQTL score
eGFRCys: % change per 1SD change in eQTL score
ACR: % change per 1SD change in eQTL score

Table S3. Two-sample summary-level Mendelian randomization analyses in CKDGen and FinnGen

						Inverse variance weighted				Instrument Strength	
**consortium**	**outcome**	**Cases**	**Controls**	**n**	**nsnp**	**OR/Beta**	**Lower 95%CI**	**Upper 95%CI**	***P***	**R^2**	**F statistic**
FinnGen	CKD	2,709	175,566		12	0.93	0.74	1.18	0.55	0.015	329
Wuttke et al.	CKD	41,395	439,303		10	0.91	0.81	1.02	0.09	0.013	297
Wuttke et al.	eGFRcrea			522,093	10	0.0014	-0.0046	0.0074	0.65	0.013	297
Wuttke et al.	UACR			522,093	11	0.02	-0.02	0.05	0.33	0.014	306
Stanzick et al.	eGFRcrea			1,201,930	8	-0.00053	-0.0053	0.0043	0.83	0.013	281
Stanzick et al.	eGFRcys			1,201,930	8	-0.00095	-0.0064	0.0045	0.73	0.013	281
Gorski et al.	CKDi25	19,901	175,244		11	1.04	0.89	1.22	0.58	0.014	311
Gorski et al.	Rapid3	34,874	107,090		8	0.88	0.76	1.01	0.07	0.013	281

						Weighted median			
**consortium**	**outcome**	**Cases**	**Controls**	**n**	**nsnp**	**OR/Beta**	**Lower 95%CI**	**Upper 95%CI**	***P***
FinnGen	CKD	2,709	175,566		12	0.96	0.73	1.27	0.77
Wuttke et al.	CKD	41,395	439,303		10	0.93	0.82	1.04	0.21
Wuttke et al.	eGFRcrea			522,093	10	-0.0011	-0.0055	0.0033	0.63
Wuttke et al.	UACR			522,093	11	0.014	-0.01	0.04	0.31
Stanzick et al.	eGFRcrea			1,201,930	8	0.0007	-0.0023	0.0037	0.65
Stanzick et al.	eGFRcys			1,201,930	8	-0.00053	-0.0064	0.0054	0.86
Gorski et al.	CKDi25	19,901	175,244		11	1.06	0.90	1.27	0.47
Gorski et al.	Rapid3	34,874	107,090		8	0.88	0.78	1.00	0.05

						Egger				
**consortium**	**outcome**	**Cases**	**Controls**	**n**	**nsnp**	**OR/Beta**	**Lower 95%CI**	**Upper 95%CI**	***P***	**Intercept P**
FinnGen	CKD	2,709	175,566		12	0.926	0.658	1.316	0.68	0.99
Wuttke et al.	CKD	41,395	439,303		10	0.926	0.794	1.075	0.33	0.81
Wuttke et al.	eGFRcrea			522,093	10	-0.0063	-0.012059	-0.000541	0.06	0.0052
Wuttke et al.	UACR			522,093	11	0.0012	-0.0433	0.05	0.96	0.39
Stanzick et al.	eGFRcrea			1,201,930	8	0.0041	-0.0008	0.009	0.16	0.039
Stanzick et al.	eGFRcys			1,201,930	8	-0.00061	-0.00812	0.0069	0.88	0.90
Gorski et al.	CKDi25	19,901	175,244		11	1.00	0.80	1.25	0.98	0.57
Gorski et al.	Rapid3	34,874	107,090		8	0.88	0.72	1.09	0.28	0.95

* Beta for CKD: OR per 1SD change in eQTL score; Beta for eGFRCrea, eGFRCys, ACR: % change per 1SD change in eQTL score

Table S4. Rare variant association results for UK biobank

Rare variants predicted to be loss-of-function

phenotype	cases	controls	n	Beta	SE	Odds ratio	Lower 95%CI	Upper 95%CI	*P*
LDL cholesterol			154220	0.013	0.12		-0.227	0.25	0.91
C reactive protein			154135	1.39	0.61		0.194	2.59	0.02
CKD	9982	152233		0.16	0.54	1.17	0.41	3.36	0.77
eGFR_crea_CKDEpi			154385	-0.25	1.67		-3.51	3.02	0.88
eGFR_cys_CKDEpi			154464	-3.74	1.96		-7.58	0.096	0.06
Urine ACR			157528	0.024	0.11		-0.182	0.23	0.82

Rare variants with Mendelian Clinical Applicable Pathogenicity (M-CAP) Score > 0.025

phenotype	cases	controls	n	Beta	SE	Odds ratio	Lower 95%CI	Upper 95%CI	*P*
LDL cholesterol			154220	-0.012	0.02		-0.052	0.027	0.54
C reactive protein			154135	-0.14	0.1		-0.34	0.056	0.16
CKD	9982	152233		-0.077	0.099	0.93	0.76	1.12	0.44
eGFR_crea_CKDEpi			154385	0.15	0.27		-0.39	0.68	0.59
eGFR_cys_CKDEpi			154464	0.32	0.32		-0.31	0.95	0.32
Urine ACR			157528	-0.0023	0.017		-0.035	0.03	0.89

*See Jagadeesh KA et al., Nat Genet 2016 for details on M-CAP
CKD defined as CKD G3, G4, G5 or eGFRCrea or eGFRCys < 60 ml/min/1.73m2
* Beta represents:
ApoB: g/L per 1 SD change in eQTL score
LDL: mmol/L per 1 SD change in eQTL score
CRP: mg/L per 1 SD change in eQTL score
CKD: OR per 1SD change in eQTL score
eGFRCr: % change per 1SD change in eQTL score
eGFRCys: % change per 1SD change in eQTL score
ACR: % change per 1SD change in eQTL score

Table S5. Predicted loss-of-function ACLY rare variants observed in UK biobank participants.

variant ID	chro	start (hg38)	end (hg38)	referen	alte	dbSNP ident	consequence
17:41868726:I:1	17	41868726	41868726	-	T	NA	frameshift insertion
17:41869487:D:1	17	41869488	41869488	T	-	NA	frameshift deletion
17:41869569:G:A	17	41869569	41869569	G	A	rs782631078	stopgain
17:41871700:G:A	17	41871700	41871700	G	A	NA	stopgain
17:41872082:G:A	17	41872082	41872082	G	A	NA	stopgain
17:41872153:I:2	17	41872153	41872153	-	CA	NA	frameshift insertion
17:41872184:T:G	17	41872184	41872184	T	G	NA	splice variant
17:41878165:I:2	17	41878165	41878165	-	CT	NA	frameshift insertion
17:41878166:D:2	17	41878167	41878168	CC	-	NA	frameshift deletion
17:41878796:C:T	17	41878796	41878796	C	T	NA	splice variant
17:41883217:C:A	17	41883217	41883217	C	A	NA	stopgain
17:41883229:D:1	17	41883230	41883230	A	-	NA	frameshift deletion
17:41884240:I:1	17	41884240	41884240	-	T	NA	frameshift insertion
17:41892420:C:T	17	41892420	41892420	C	T	NA	stopgain
17:41893031:A:C	17	41893031	41893031	A	C	NA	splice variant
17:41893031:D:1	17	41893032	41893032	C	-	rs782813736	splice variant
17:41893073:G:A	17	41893073	41893073	G	A	rs944249979	stopgain
17:41897749:C:A	17	41897749	41897749	C	A	NA	stopgain
17:41897840:C:T	17	41897840	41897840	C	T	NA	splice variant
17:41901795:G:A	17	41901795	41901795	G	A	rs918567524	stopgain
17:41909565:D:2	17	41909566	41909567	CA	-	NA	frameshift deletion
17:41909680:D:4	17	41909681	41909684	CAGA	-	NA	frameshift deletion
17:41910229:D:1	17	41910230	41910230	G	-	NA	frameshift deletion
17:41913717:G:A	17	41913717	41913717	G	A	NA	stopgain
17:41913873:T:C	17	41913873	41913873	T	C	NA	startloss
17:41913879:D:2	17	41913880	41913881	AG	-	NA	frameshift deletion
17:41918937:I:2	17	41918937	41918937	-	CG	NA	frameshift insertion
17:41918947:D:11	17	41918948	41918958	CAGCA	-	NA	frameshift deletion
17:41918996:D:1	17	41918997	41918997	A	-	NA	frameshift deletion
17:41919002:T:A	17	41919002	41919002	T	A	NA	stopgain

Table S6. Predicted deleterious ACLY rare variants observed in UK biobank participants.
Rare variants with Mendelian Clinical Applicable Pathogenicity (M-CAP) Score > 0.025

variant ID	chrc	start (hg38)	end (hg38)	refe	alte	dbSNP identi	consequence
17:41867817:C:A	17	41867817	41867817	C	A	NA	nonsynonymous SNV
17:41867821:T:G	17	41867821	41867821	T	G	NA	nonsynonymous SNV
17:41867829:G:A	17	41867829	41867829	G	A	rs148640026	nonsynonymous SNV
17:41867829:G:T	17	41867829	41867829	G	T	NA	nonsynonymous SNV
17:41867835:A:C	17	41867835	41867835	A	C	NA	nonsynonymous SNV
17:41867862:C:T	17	41867862	41867862	C	T	NA	nonsynonymous SNV
17:41867872:C:T	17	41867872	41867872	C	T	NA	nonsynonymous SNV
17:41867896:G:C	17	41867896	41867896	G	C	NA	nonsynonymous SNV
17:41868718:C:A	17	41868718	41868718	C	A	NA	nonsynonymous SNV
17:41868757:C:T	17	41868757	41868757	C	T	NA	nonsynonymous SNV
17:41868759:A:G	17	41868759	41868759	A	G	rs782304822	nonsynonymous SNV
17:41868760:T:C	17	41868760	41868760	T	C	NA	nonsynonymous SNV
17:41868771:T:C	17	41868771	41868771	T	C	NA	nonsynonymous SNV
17:41869043:C:T	17	41869043	41869043	C	T	NA	nonsynonymous SNV
17:41869046:G:A	17	41869046	41869046	G	A	rs782134690	nonsynonymous SNV
17:41869067:A:G	17	41869067	41869067	A	G	NA	nonsynonymous SNV
17:41869073:T:C	17	41869073	41869073	T	C	NA	nonsynonymous SNV
17:41869083:C:T	17	41869083	41869083	C	T	rs376874300	nonsynonymous SNV
17:41869092:T:C	17	41869092	41869092	T	C	rs1007013795	nonsynonymous SNV
17:41869115:A:C	17	41869115	41869115	A	C	NA	nonsynonymous SNV
17:41869119:T:C	17	41869119	41869119	T	C	NA	nonsynonymous SNV
17:41869123:C:G	17	41869123	41869123	C	G	NA	nonsynonymous SNV
17:41869486:A:C	17	41869486	41869486	A	C	NA	nonsynonymous SNV
17:41869500:C:T	17	41869500	41869500	C	T	rs898646965	nonsynonymous SNV
17:41869512:C:T	17	41869512	41869512	C	T	NA	nonsynonymous SNV
17:41869515:G:C	17	41869515	41869515	G	C	rs782778188	nonsynonymous SNV
17:41869521:G:A	17	41869521	41869521	G	A	rs781890934	nonsynonymous SNV
17:41869527:C:T	17	41869527	41869527	C	T	NA	nonsynonymous SNV
17:41869544:A:G	17	41869544	41869544	A	G	NA	nonsynonymous SNV
17:41869545:C:T	17	41869545	41869545	C	T	NA	nonsynonymous SNV
17:41869550:T:A	17	41869550	41869550	T	A	rs1026333389	nonsynonymous SNV
17:41869553:T:C	17	41869553	41869553	T	C	NA	nonsynonymous SNV
17:41869568:C:T	17	41869568	41869568	C	T	NA	nonsynonymous SNV
17:41869571:A:G	17	41869571	41869571	A	G	NA	nonsynonymous SNV
17:41871699:C:T	17	41871699	41871699	C	T	NA	nonsynonymous SNV
17:41871737:C:A	17	41871737	41871737	C	A	rs782637582	nonsynonymous SNV
17:41871738:A:G	17	41871738	41871738	A	G	rs139693539	nonsynonymous SNV
17:41871798:G:A	17	41871798	41871798	G	A	NA	nonsynonymous SNV
17:41871807:T:C	17	41871807	41871807	T	C	rs1048697464	nonsynonymous SNV
17:41871814:C:A	17	41871814	41871814	C	A	NA	nonsynonymous SNV

17:41871821:A:T	17	41871821	41871821	A	T	NA	nonsynonymous SNV
17:41871825:C:T	17	41871825	41871825	C	T	rs782319132	nonsynonymous SNV
17:41872042:A:G	17	41872042	41872042	A	G	NA	nonsynonymous SNV
17:41872052:T:C	17	41872052	41872052	T	C	NA	nonsynonymous SNV
17:41872055:G:A	17	41872055	41872055	G	A	NA	nonsynonymous SNV
17:41872064:C:A	17	41872064	41872064	C	A	NA	nonsynonymous SNV
17:41872072:T:C	17	41872072	41872072	T	C	NA	nonsynonymous SNV
17:41872079:C:T	17	41872079	41872079	C	T	NA	nonsynonymous SNV
17:41872084:G:A	17	41872084	41872084	G	A	rs375650392	nonsynonymous SNV
17:41872097:T:C	17	41872097	41872097	T	C	rs782205140	nonsynonymous SNV
17:41872106:C:A	17	41872106	41872106	C	A	NA	nonsynonymous SNV
17:41872114:A:G	17	41872114	41872114	A	G	NA	nonsynonymous SNV
17:41872132:G:C	17	41872132	41872132	G	C	NA	nonsynonymous SNV
17:41872140:C:G	17	41872140	41872140	C	G	rs370553090	nonsynonymous SNV
17:41872142:T:C	17	41872142	41872142	T	C	NA	nonsynonymous SNV
17:41872174:T:G	17	41872174	41872174	T	G	NA	nonsynonymous SNV
17:41872178:G:A	17	41872178	41872178	G	A	NA	nonsynonymous SNV
17:41873822:C:G	17	41873822	41873822	C	G	NA	nonsynonymous SNV
17:41873842:C:T	17	41873842	41873842	C	T	NA	nonsynonymous SNV
17:41873877:A:G	17	41873877	41873877	A	G	NA	nonsynonymous SNV
17:41873886:C:T	17	41873886	41873886	C	T	rs782376333	nonsynonymous SNV
17:41873904:T:C	17	41873904	41873904	T	C	NA	nonsynonymous SNV
17:41878126:G:T	17	41878126	41878126	G	T	NA	nonsynonymous SNV
17:41878129:C:T	17	41878129	41878129	C	T	NA	nonsynonymous SNV
17:41878147:C:T	17	41878147	41878147	C	T	NA	nonsynonymous SNV
17:41878161:A:G	17	41878161	41878161	A	G	NA	nonsynonymous SNV
17:41878813:G:A	17	41878813	41878813	G	A	NA	nonsynonymous SNV
17:41878842:C:T	17	41878842	41878842	C	T	NA	nonsynonymous SNV
17:41878912:G:A	17	41878912	41878912	G	A	NA	nonsynonymous SNV
17:41883122:C:G	17	41883122	41883122	C	G	rs782588191	nonsynonymous SNV
17:41883125:D:3	17	41883126	41883128	GA(	-	NA	nonframeshift deletion
17:41883147:G:A	17	41883147	41883147	G	A	rs782263566	nonsynonymous SNV
17:41883166:C:T	17	41883166	41883166	C	T	rs782677992	nonsynonymous SNV
17:41883171:G:A	17	41883171	41883171	G	A	NA	nonsynonymous SNV
17:41883180:A:G	17	41883180	41883180	A	G	NA	nonsynonymous SNV
17:41883196:T:A	17	41883196	41883196	T	A	NA	nonsynonymous SNV
17:41883222:G:C	17	41883222	41883222	G	C	NA	nonsynonymous SNV
17:41883231:A:G	17	41883231	41883231	A	G	rs201502123	nonsynonymous SNV
17:41884209:A:G	17	41884209	41884209	A	G	NA	nonsynonymous SNV
17:41884236:T:C	17	41884236	41884236	T	C	rs782683248	nonsynonymous SNV
17:41884239:C:T	17	41884239	41884239	C	T	NA	nonsynonymous SNV
17:41884240:G:A	17	41884240	41884240	G	A	rs782444196	nonsynonymous SNV
17:41884240:G:T	17	41884240	41884240	G	T	rs782444196	nonsynonymous SNV
17:41884241:T:A	17	41884241	41884241	T	A	NA	nonsynonymous SNV
17:41884249:G:A	17	41884249	41884249	G	A	NA	nonsynonymous SNV
17:41884257:A:C	17	41884257	41884257	A	C	NA	nonsynonymous SNV
17:41886121:C:A	17	41886121	41886121	C	A	NA	nonsynonymous SNV

17:41886124:A:G	17	41886124	41886124	A	G	rs782010539	nonsynonymous SNV
17:41886131:C:T	17	41886131	41886131	C	T	NA	nonsynonymous SNV
17:41886139:T:G	17	41886139	41886139	T	G	NA	nonsynonymous SNV
17:41886143:C:T	17	41886143	41886143	C	T	rs201998406	nonsynonymous SNV
17:41886151:G:A	17	41886151	41886151	G	A	rs571420153	nonsynonymous SNV
17:41886157:C:T	17	41886157	41886157	C	T	NA	nonsynonymous SNV
17:41886158:G:A	17	41886158	41886158	G	A	rs141606581	nonsynonymous SNV
17:41886158:G:C	17	41886158	41886158	G	C	rs141606581	nonsynonymous SNV
17:41886169:T:C	17	41886169	41886169	T	C	NA	nonsynonymous SNV
17:41886179:C:T	17	41886179	41886179	C	T	NA	nonsynonymous SNV
17:41886188:T:C	17	41886188	41886188	T	C	rs782644505	nonsynonymous SNV
17:41886190:C:G	17	41886190	41886190	C	G	NA	nonsynonymous SNV
17:41886194:C:T	17	41886194	41886194	C	T	NA	nonsynonymous SNV
17:41886199:C:T	17	41886199	41886199	C	T	rs782573989	nonsynonymous SNV
17:41886200:G:A	17	41886200	41886200	G	A	rs538785849	nonsynonymous SNV
17:41886217:C:T	17	41886217	41886217	C	T	NA	nonsynonymous SNV
17:41886220:C:G	17	41886220	41886220	C	G	NA	nonsynonymous SNV
17:41886224:G:A	17	41886224	41886224	G	A	NA	nonsynonymous SNV
17:41886226:C:T	17	41886226	41886226	C	T	rs782018591	nonsynonymous SNV
17:41886227:G:A	17	41886227	41886227	G	A	rs565573136	nonsynonymous SNV
17:41886234:T:A	17	41886234	41886234	T	A	rs782696154	nonsynonymous SNV
17:41886258:C:T	17	41886258	41886258	C	T	NA	nonsynonymous SNV
17:41886286:C:T	17	41886286	41886286	C	T	NA	nonsynonymous SNV
17:41886289:C:T	17	41886289	41886289	C	T	NA	nonsynonymous SNV
17:41886297:G:C	17	41886297	41886297	G	C	rs781783769	nonsynonymous SNV
17:41886298:A:G	17	41886298	41886298	A	G	NA	nonsynonymous SNV
17:41887613:T:C	17	41887613	41887613	T	C	rs782458381	nonsynonymous SNV
17:41887615:A:C	17	41887615	41887615	A	C	rs782598021	nonsynonymous SNV
17:41887624:C:T	17	41887624	41887624	C	T	NA	nonsynonymous SNV
17:41887633:T:A	17	41887633	41887633	T	A	NA	nonsynonymous SNV
17:41887636:G:A	17	41887636	41887636	G	A	rs142738500	nonsynonymous SNV
17:41887637:C:T	17	41887637	41887637	C	T	NA	nonsynonymous SNV
17:41887645:A:G	17	41887645	41887645	A	G	NA	nonsynonymous SNV
17:41887649:G:T	17	41887649	41887649	G	T	rs781983403	nonsynonymous SNV
17:41887651:T:C	17	41887651	41887651	T	C	rs782788584	nonsynonymous SNV
17:41887657:G:A	17	41887657	41887657	G	A	NA	nonsynonymous SNV
17:41887657:G:C	17	41887657	41887657	G	C	NA	nonsynonymous SNV
17:41887685:T:C	17	41887685	41887685	T	C	rs781831373	nonsynonymous SNV
17:41887690:G:A	17	41887690	41887690	G	A	NA	nonsynonymous SNV
17:41887691:C:T	17	41887691	41887691	C	T	rs946866792	nonsynonymous SNV
17:41887696:G:A	17	41887696	41887696	G	A	rs782649298	nonsynonymous SNV
17:41887699:C:A	17	41887699	41887699	C	A	NA	nonsynonymous SNV
17:41887699:C:T	17	41887699	41887699	C	T	rs147387411	nonsynonymous SNV
17:41887700:G:A	17	41887700	41887700	G	A	rs537907558	nonsynonymous SNV
17:41887700:G:C	17	41887700	41887700	G	C	NA	nonsynonymous SNV
17:41892293:T:C	17	41892293	41892293	T	C	NA	nonsynonymous SNV
17:41892313:T:C	17	41892313	41892313	T	C	rs763074358	nonsynonymous SNV

17:41892323:G:A	17	41892323	41892323	G	A	NA	nonsynonymous SNV
17:41892326:G:A	17	41892326	41892326	G	A	NA	nonsynonymous SNV
17:41892346:A:G	17	41892346	41892346	A	G	NA	nonsynonymous SNV
17:41892358:G:A	17	41892358	41892358	G	A	rs781816445	nonsynonymous SNV
17:41892373:G:A	17	41892373	41892373	G	A	NA	nonsynonymous SNV
17:41892374:C:T	17	41892374	41892374	C	T	NA	nonsynonymous SNV
17:41892377:C:G	17	41892377	41892377	C	G	NA	nonsynonymous SNV
17:41892377:C:T	17	41892377	41892377	C	T	NA	nonsynonymous SNV
17:41892379:G:A	17	41892379	41892379	G	A	rs368294825	nonsynonymous SNV
17:41892383:T:C	17	41892383	41892383	T	C	NA	nonsynonymous SNV
17:41892385:T:C	17	41892385	41892385	T	C	rs965778951	nonsynonymous SNV
17:41892390:G:T	17	41892390	41892390	G	T	NA	nonsynonymous SNV
17:41892392:A:G	17	41892392	41892392	A	G	rs532453858	nonsynonymous SNV
17:41892392:A:T	17	41892392	41892392	A	T	NA	nonsynonymous SNV
17:41892401:T:C	17	41892401	41892401	T	C	rs781869662	nonsynonymous SNV
17:41892419:C:T	17	41892419	41892419	C	T	NA	nonsynonymous SNV
17:41892427:A:C	17	41892427	41892427	A	C	NA	nonsynonymous SNV
17:41893033:G:A	17	41893033	41893033	G	A	NA	nonsynonymous SNV
17:41893035:G:T	17	41893035	41893035	G	T	NA	nonsynonymous SNV
17:41893045:A:G	17	41893045	41893045	A	G	NA	nonsynonymous SNV
17:41893052:C:A	17	41893052	41893052	C	A	NA	nonsynonymous SNV
17:41893054:G:C	17	41893054	41893054	G	C	NA	nonsynonymous SNV
17:41893058:C:T	17	41893058	41893058	C	T	NA	nonsynonymous SNV
17:41893072:C:A	17	41893072	41893072	C	A	NA	nonsynonymous SNV
17:41893109:C:T	17	41893109	41893109	C	T	rs1041624299	nonsynonymous SNV
17:41893115:G:A	17	41893115	41893115	G	A	rs782295756	nonsynonymous SNV
17:41893133:C:T	17	41893133	41893133	C	T	rs151030814	nonsynonymous SNV
17:41893138:G:A	17	41893138	41893138	G	A	rs782086628	nonsynonymous SNV
17:41893142:T:C	17	41893142	41893142	T	C	NA	nonsynonymous SNV
17:41893150:C:T	17	41893150	41893150	C	T	rs387907386	nonsynonymous SNV
17:41893151:G:A	17	41893151	41893151	G	A	rs141255682	nonsynonymous SNV
17:41893157:A:G	17	41893157	41893157	A	G	NA	nonsynonymous SNV
17:41893163:T:C	17	41893163	41893163	T	C	NA	nonsynonymous SNV
17:41893168:C:T	17	41893168	41893168	C	T	rs782499809	nonsynonymous SNV
17:41896620:C:T	17	41896620	41896620	C	T	NA	nonsynonymous SNV
17:41896628:G:A	17	41896628	41896628	G	A	rs375642586	nonsynonymous SNV
17:41896628:G:C	17	41896628	41896628	G	C	NA	nonsynonymous SNV
17:41896632:T:C	17	41896632	41896632	T	C	NA	nonsynonymous SNV
17:41897755:G:A	17	41897755	41897755	G	A	NA	nonsynonymous SNV
17:41897757:I:18	17	41897757	41897757	-	TG[illegible]	NA	nonframeshift insertion
17:41897778:G:A	17	41897778	41897778	G	A	NA	nonsynonymous SNV
17:41897782:G:A	17	41897782	41897782	G	A	NA	nonsynonymous SNV
17:41897788:C:T	17	41897788	41897788	C	T	rs781966325	nonsynonymous SNV
17:41897800:T:C	17	41897800	41897800	T	C	NA	nonsynonymous SNV
17:41897803:A:G	17	41897803	41897803	A	G	rs782159286	nonsynonymous SNV
17:41898632:G:A	17	41898632	41898632	G	A	rs146823754	nonsynonymous SNV
17:41898642:C:T	17	41898642	41898642	C	T	rs61749881	nonsynonymous SNV

17:41898648:C:T	17	41898648	41898648	C	T	rs999256485	nonsynonymous SNV
17:41898665:G:A	17	41898665	41898665	G	A	NA	nonsynonymous SNV
17:41898677:G:A	17	41898677	41898677	G	A	rs782383157	nonsynonymous SNV
17:41898704:C:T	17	41898704	41898704	C	T	rs781923056	nonsynonymous SNV
17:41898705:G:A	17	41898705	41898705	G	A	NA	nonsynonymous SNV
17:41898718:C:G	17	41898718	41898718	C	G	NA	nonsynonymous SNV
17:41898726:C:T	17	41898726	41898726	C	T	NA	nonsynonymous SNV
17:41898732:C:G	17	41898732	41898732	C	G	NA	nonsynonymous SNV
17:41898734:G:A	17	41898734	41898734	G	A	rs782419889	nonsynonymous SNV
17:41898773:C:T	17	41898773	41898773	C	T	NA	nonsynonymous SNV
17:41898774:C:T	17	41898774	41898774	C	T	rs782065012	nonsynonymous SNV
17:41898777:T:G	17	41898777	41898777	T	G	rs782102822	nonsynonymous SNV
17:41901701:T:G	17	41901701	41901701	T	G	NA	nonsynonymous SNV
17:41901707:A:C	17	41901707	41901707	A	C	NA	nonsynonymous SNV
17:41901714:G:A	17	41901714	41901714	G	A	rs782729236	nonsynonymous SNV
17:41901735:G:A	17	41901735	41901735	G	A	NA	nonsynonymous SNV
17:41901780:G:A	17	41901780	41901780	G	A	NA	nonsynonymous SNV
17:41901791:T:A	17	41901791	41901791	T	A	NA	nonsynonymous SNV
17:41904748:T:C	17	41904748	41904748	T	C	NA	nonsynonymous SNV
17:41904779:T:C	17	41904779	41904779	T	C	rs782705078	nonsynonymous SNV
17:41905522:C:T	17	41905522	41905522	C	T	NA	nonsynonymous SNV
17:41905524:T:A	17	41905524	41905524	T	A	NA	nonsynonymous SNV
17:41905529:G:C	17	41905529	41905529	G	C	rs781817246	nonsynonymous SNV
17:41905546:T:C	17	41905546	41905546	T	C	NA	nonsynonymous SNV
17:41905555:G:C	17	41905555	41905555	G	C	rs782628207	nonsynonymous SNV
17:41905558:T:C	17	41905558	41905558	T	C	NA	nonsynonymous SNV
17:41905584:T:C	17	41905584	41905584	T	C	NA	nonsynonymous SNV
17:41905588:C:T	17	41905588	41905588	C	T	rs200778231	nonsynonymous SNV
17:41905597:C:T	17	41905597	41905597	C	T	NA	nonsynonymous SNV
17:41905611:C:T	17	41905611	41905611	C	T	NA	nonsynonymous SNV
17:41905616:G:C	17	41905616	41905616	G	C	NA	nonsynonymous SNV
17:41905617:T:G	17	41905617	41905617	T	G	NA	nonsynonymous SNV
17:41905627:C:T	17	41905627	41905627	C	T	NA	nonsynonymous SNV
17:41905635:C:A	17	41905635	41905635	C	A	NA	nonsynonymous SNV
17:41905638:C:T	17	41905638	41905638	C	T	NA	nonsynonymous SNV
17:41905645:C:G	17	41905645	41905645	C	G	NA	nonsynonymous SNV
17:41905647:C:A	17	41905647	41905647	C	A	NA	nonsynonymous SNV
17:41906528:C:T	17	41906528	41906528	C	T	NA	nonsynonymous SNV
17:41906535:C:T	17	41906535	41906535	C	T	rs369798437	nonsynonymous SNV
17:41906550:C:T	17	41906550	41906550	C	T	NA	nonsynonymous SNV
17:41906559:C:A	17	41906559	41906559	C	A	NA	nonsynonymous SNV
17:41906560:C:A	17	41906560	41906560	C	A	NA	nonsynonymous SNV
17:41906562:T:C	17	41906562	41906562	T	C	rs782069282	nonsynonymous SNV
17:41906571:T:G	17	41906571	41906571	T	G	NA	nonsynonymous SNV
17:41906600:T:C	17	41906600	41906600	T	C	NA	nonsynonymous SNV
17:41906633:G:A	17	41906633	41906633	G	A	NA	nonsynonymous SNV
17:41907458:C:T	17	41907458	41907458	C	T	rs183942395	nonsynonymous SNV

17:41907459:G:A	17	41907459	41907459	G	A	NA	nonsynonymous SNV
17:41907461:C:A	17	41907461	41907461	C	A	NA	nonsynonymous SNV
17:41907467:G:A	17	41907467	41907467	G	A	rs150561917	nonsynonymous SNV
17:41907467:G:C	17	41907467	41907467	G	C	rs150561917	nonsynonymous SNV
17:41907467:G:T	17	41907467	41907467	G	T	rs150561917	nonsynonymous SNV
17:41907468:G:C	17	41907468	41907468	G	C	NA	nonsynonymous SNV
17:41907474:G:A	17	41907474	41907474	G	A	rs781915514	nonsynonymous SNV
17:41907504:A:T	17	41907504	41907504	A	T	NA	nonsynonymous SNV
17:41907513:C:T	17	41907513	41907513	C	T	rs782737276	nonsynonymous SNV
17:41907525:C:T	17	41907525	41907525	C	T	NA	nonsynonymous SNV
17:41907536:G:A	17	41907536	41907536	G	A	rs139584437	nonsynonymous SNV
17:41907537:C:T	17	41907537	41907537	C	T	rs387907387	nonsynonymous SNV
17:41907551:T:C	17	41907551	41907551	T	C	rs200766140	nonsynonymous SNV
17:41908989:C:T	17	41908989	41908989	C	T	NA	nonsynonymous SNV
17:41909004:C:T	17	41909004	41909004	C	T	rs782631257	nonsynonymous SNV
17:41909024:T:C	17	41909024	41909024	T	C	NA	nonsynonymous SNV
17:41909028:C:T	17	41909028	41909028	C	T	rs576161746	nonsynonymous SNV
17:41909046:C:T	17	41909046	41909046	C	T	NA	nonsynonymous SNV
17:41909055:A:T	17	41909055	41909055	A	T	NA	nonsynonymous SNV
17:41909528:G:C	17	41909528	41909528	G	C	rs781944768	nonsynonymous SNV
17:41909529:C:A	17	41909529	41909529	C	A	rs372205779	nonsynonymous SNV
17:41909529:C:T	17	41909529	41909529	C	T	rs372205779	nonsynonymous SNV
17:41909532:G:C	17	41909532	41909532	G	C	NA	nonsynonymous SNV
17:41909549:T:A	17	41909549	41909549	T	A	rs782131682	nonsynonymous SNV
17:41909556:C:A	17	41909556	41909556	C	A	rs782758020	nonsynonymous SNV
17:41909556:C:T	17	41909556	41909556	C	T	NA	nonsynonymous SNV
17:41909577:C:T	17	41909577	41909577	C	T	NA	nonsynonymous SNV
17:41909580:C:G	17	41909580	41909580	C	G	NA	nonsynonymous SNV
17:41909580:C:T	17	41909580	41909580	C	T	NA	nonsynonymous SNV
17:41909583:C:G	17	41909583	41909583	C	G	NA	nonsynonymous SNV
17:41909586:C:A	17	41909586	41909586	C	A	NA	nonsynonymous SNV
17:41909619:C:T	17	41909619	41909619	C	T	NA	nonsynonymous SNV
17:41909622:C:T	17	41909622	41909622	C	T	rs782606504	nonsynonymous SNV
17:41909633:C:A	17	41909633	41909633	C	A	NA	nonsynonymous SNV
17:41909633:C:G	17	41909633	41909633	C	G	rs782037288	nonsynonymous SNV
17:41909634:C:A	17	41909634	41909634	C	A	NA	nonsynonymous SNV
17:41909635:C:A	17	41909635	41909635	C	A	rs782151031	nonsynonymous SNV
17:41909635:C:G	17	41909635	41909635	C	G	NA	nonsynonymous SNV
17:41909636:T:C	17	41909636	41909636	T	C	NA	nonsynonymous SNV
17:41909637:C:T	17	41909637	41909637	C	T	rs782128270	nonsynonymous SNV
17:41909666:C:T	17	41909666	41909666	C	T	rs140011228	nonsynonymous SNV
17:41909669:G:A	17	41909669	41909669	G	A	NA	nonsynonymous SNV
17:41909675:T:C	17	41909675	41909675	T	C	rs558283153	nonsynonymous SNV
17:41910223:T:C	17	41910223	41910223	T	C	NA	nonsynonymous SNV
17:41910230:G:T	17	41910230	41910230	G	T	NA	nonsynonymous SNV
17:41910245:C:T	17	41910245	41910245	C	T	rs1048505068	nonsynonymous SNV
17:41910246:G:C	17	41910246	41910246	G	C	rs782487022	nonsynonymous SNV

17:41910260:T:C	17	41910260	41910260	T	C	NA	nonsynonymous SNV
17:41910268:C:G	17	41910268	41910268	C	G	NA	nonsynonymous SNV
17:41912421:G:A	17	41912421	41912421	G	A	rs782275584	nonsynonymous SNV
17:41912424:G:T	17	41912424	41912424	G	T	rs376094175	nonsynonymous SNV
17:41912439:C:T	17	41912439	41912439	C	T	rs574241105	nonsynonymous SNV
17:41912440:G:A	17	41912440	41912440	G	A	rs41280076	nonsynonymous SNV
17:41912454:G:A	17	41912454	41912454	G	A	rs782047538	nonsynonymous SNV
17:41912478:T:A	17	41912478	41912478	T	A	rs781834702	nonsynonymous SNV
17:41912478:T:C	17	41912478	41912478	T	C	rs781834702	nonsynonymous SNV
17:41912478:T:G	17	41912478	41912478	T	G	rs781834702	nonsynonymous SNV
17:41912485:C:G	17	41912485	41912485	C	G	NA	nonsynonymous SNV
17:41912487:A:G	17	41912487	41912487	A	G	NA	nonsynonymous SNV
17:41912488:C:A	17	41912488	41912488	C	A	NA	nonsynonymous SNV
17:41912488:C:T	17	41912488	41912488	C	T	NA	nonsynonymous SNV
17:41912496:A:G	17	41912496	41912496	A	G	NA	nonsynonymous SNV
17:41912502:C:A	17	41912502	41912502	C	A	NA	nonsynonymous SNV
17:41912505:C:T	17	41912505	41912505	C	T	rs782250367	nonsynonymous SNV
17:41912509:G:A	17	41912509	41912509	G	A	rs782360379	nonsynonymous SNV
17:41912520:T:C	17	41912520	41912520	T	C	rs782309371	nonsynonymous SNV
17:41913732:G:T	17	41913732	41913732	G	T	NA	nonsynonymous SNV
17:41913754:C:A	17	41913754	41913754	C	A	NA	nonsynonymous SNV
17:41913756:A:G	17	41913756	41913756	A	G	NA	nonsynonymous SNV
17:41913771:T:C	17	41913771	41913771	T	C	NA	nonsynonymous SNV
17:41913777:G:A	17	41913777	41913777	G	A	rs148951948	nonsynonymous SNV
17:41913782:T:C	17	41913782	41913782	T	C	rs782539870	nonsynonymous SNV
17:41913792:G:A	17	41913792	41913792	G	A	NA	nonsynonymous SNV
17:41913800:A:G	17	41913800	41913800	A	G	NA	nonsynonymous SNV
17:41913809:G:A	17	41913809	41913809	G	A	rs782215204	nonsynonymous SNV
17:41913813:T:C	17	41913813	41913813	T	C	rs782688599	nonsynonymous SNV
17:41913845:G:A	17	41913845	41913845	G	A	rs200879681	nonsynonymous SNV
17:41913848:T:C	17	41913848	41913848	T	C	NA	nonsynonymous SNV
17:41913864:T:C	17	41913864	41913864	T	C	NA	nonsynonymous SNV
17:41913878:G:A	17	41913878	41913878	G	A	rs371523591	nonsynonymous SNV
17:41913899:G:A	17	41913899	41913899	G	A	NA	nonsynonymous SNV
17:41918884:G:A	17	41918884	41918884	G	A	rs772582665	nonsynonymous SNV
17:41918887:G:A	17	41918887	41918887	G	A	NA	nonsynonymous SNV
17:41918887:G:C	17	41918887	41918887	G	C	rs782117142	nonsynonymous SNV
17:41918894:G:A	17	41918894	41918894	G	A	NA	nonsynonymous SNV
17:41918899:C:A	17	41918899	41918899	C	A	NA	nonsynonymous SNV
17:41918903:G:C	17	41918903	41918903	G	C	rs782129290	nonsynonymous SNV
17:41918911:C:A	17	41918911	41918911	C	A	NA	nonsynonymous SNV
17:41918917:C:A	17	41918917	41918917	C	A	NA	nonsynonymous SNV
17:41918917:C:T	17	41918917	41918917	C	T	NA	nonsynonymous SNV
17:41918920:G:C	17	41918920	41918920	G	C	NA	nonsynonymous SNV
17:41918923:G:A	17	41918923	41918923	G	A	rs387907380	nonsynonymous SNV
17:41918930:G:A	17	41918930	41918930	G	A	rs1016218608	nonsynonymous SNV
17:41918935:A:G	17	41918935	41918935	A	G	rs531158738	nonsynonymous SNV

17:41918935:A:T	17	41918935	41918935	A	T	NA	nonsynonymous SNV
17:41918936:T:A	17	41918936	41918936	T	A	NA	nonsynonymous SNV
17:41918936:T:G	17	41918936	41918936	T	G	NA	nonsynonymous SNV
17:41918938:C:A	17	41918938	41918938	C	A	NA	nonsynonymous SNV
17:41918938:C:T	17	41918938	41918938	C	T	NA	nonsynonymous SNV
17:41918939:G:A	17	41918939	41918939	G	A	rs782655240	nonsynonymous SNV
17:41918942:G:A	17	41918942	41918942	G	A	NA	nonsynonymous SNV
17:41918942:G:C	17	41918942	41918942	G	C	NA	nonsynonymous SNV
17:41918945:C:A	17	41918945	41918945	C	A	NA	nonsynonymous SNV
17:41918951:C:G	17	41918951	41918951	C	G	NA	nonsynonymous SNV
17:41918953:G:A	17	41918953	41918953	G	A	rs972568059	nonsynonymous SNV
17:41918962:T:G	17	41918962	41918962	T	G	NA	nonsynonymous SNV
17:41918966:C:A	17	41918966	41918966	C	A	NA	nonsynonymous SNV
17:41918966:C:G	17	41918966	41918966	C	G	NA	nonsynonymous SNV
17:41918969:G:A	17	41918969	41918969	G	A	rs546727022	nonsynonymous SNV
17:41918972:C:T	17	41918972	41918972	C	T	NA	nonsynonymous SNV
17:41918974:G:A	17	41918974	41918974	G	A	rs568070855	nonsynonymous SNV
17:41918978:T:G	17	41918978	41918978	T	G	NA	nonsynonymous SNV
17:41918983:C:A	17	41918983	41918983	C	A	rs782194209	nonsynonymous SNV
17:41918983:C:T	17	41918983	41918983	C	T	NA	nonsynonymous SNV
17:41918986:G:C	17	41918986	41918986	G	C	rs936312560	nonsynonymous SNV
17:41918993:C:G	17	41918993	41918993	C	G	NA	nonsynonymous SNV
17:41918993:C:T	17	41918993	41918993	C	T	NA	nonsynonymous SNV
17:41919002:T:C	17	41919002	41919002	T	C	NA	nonsynonymous SNV

STROBE Checklist for "ATP-citrate lyase as a therapeutic target in chronic kidney disease"

#	Section(s)	Item	Section and Page No.
1	TITLE and ABSTRACT	Indicate Mendelian randomization as the study's design in the title and/or abst	Title page and abstract
2	INTRODUCTION: Background	Explain the scientific background and rationale for the reported study. Is causality between exposure and outcome plausible? Justify why MR is a helpful method to address the study question.	Introduction
3	INTRODUCTION: Objectives	State specific objectives clearly, including pre-specified causal hypotheses (if an	Introduction
4	METHODS: Study Design + Data Sourc	Present key elements of study design early in the paper. Consider including a table listing sources of data for all phases of the study. For each data source contributing to the analysis, describe the following:	Methods
		a) Describe the study design and the underlying population from which it was drawn. Describe also the setting, locations, and relevant dates, including periods of recruitment, exposure, follow-up, and data collection, if available.	Methods
		b) Give the eligibility criteria, and the sources and methods of selection of participants.	Methods
		c) Explain how the analyzed sample size was arrived at.	Methods
		d) Describe measurement, quality and selection of genetic variants.	Methods
		e) For each exposure, outcome and other relevant variables, describe methods of assessment and, in the case of diseases, the diagnostic criteria used.	Methods
		f) Provide details of ethics committee approval and participant informed consent, if relevant.	NA
5	METHODS: Assumptions	Explicitly state assumptions for the main analysis (e.g. relevance, exclusion, independence, homogeneity) as well assumptions for any additional or sensitivity analysis.	Methods
6	METHODS: Statistical Methods: main a	a) Describe how quantitative variables were handled in the analyses (i.e., scale, units, model).	Methods
		b) Describe the process for identifying genetic variants and weights to be included in the analyses (i.e, independence and model). Consider a flow diagram.	Methods
		c) Describe the MR estimator, e.g. two-stage least squares, Wald ratio, and related statistics. Detail the included covariates and, in case of two-sample MR, whether the same covariate set was used for adjustment in the two samples.	Methods
		d) Explain how missing data were addressed.	Methods
		e) If applicable, say how multiple testing was dealt with.	Methods
7	METHODS: Assesment of Assumptions	Describe any methods used to assess the assumptions or justify their validity.	Methods
8	METHODS: Sensitivity Analyses	Describe any sensitivity analyses or additional analyses performed.	Methods
9	METHODS: Software & Pre-registratio	a) Name statistical software and package(s), including version and settings used	Methods
		b) State whether the study protocol and details were pre-registered (as well as when and where).	NA
10	RESULTS: Descriptive Data	a) Report the numbers of individuals at each stage of included studies and reasons for exclusion. Consider use of a flow-diagram.	Methods
		b) Report summary statistics for phenotypic exposure(s), outcome(s) and other relevant variables (e.g. means, standard deviations, proportions).	Supplementary Table 1
		c) If the data sources include meta-analyses of previous studies, provide the number of studies, their reported ancestry, if available, and assessments of heterogeneity across these studies. Consider using a supplementary table for each data source.	Supplementary Table 4
		d) For two-sample Mendelian randomization:<br>i. Provide information on the similarity of the genetic variant-exposure associations between the exposure and outcome samples.	NA
		ii. Provide information on extent of sample overlap between the exposure and outcome data sources.	Methods
11	RESULTS: Main Results	a) Report the associations between genetic variant and exposure, and between genetic variant and outcome, preferably on an interpretable scale (e.g. comparing 25th and 75th percentile of allele count or genetic risk score, if individual-level data available).	Results

		b) Report causal effect estimate between exposure and outcome, and the measures of uncertainty from the MR analysis. Use an intuitive scale, such as odds ratio, or relative risk, per standard deviation difference.	Results
		c) If relevant, consider translating estimates of relative risk into absolute risk for a meaningful time-period.	NA
		d) Consider any plots to visualize results (e.g. forest plot, scatterplot of associations between genetic variants and outcome versus between genetic variants and exposure).	Supplementary Figure
12	RESULTS: Assessment of Assumptions	a) Assess the validity of the assumptions.	Results
		b) Report any additional statistics (e.g., assessments of heterogeneity, such as I2 , Q statistic).	Results, Supplementary Table 3
13	RESULTS: Sensitivity + Additional Analy	a) Use sensitivity analyses to assess the robustness of the main results to violations of the assumptions.	Results
		b) Report results from other sensitivity analyses (e.g., replication study with different dataset, analyses of subgroups, validation of instrument(s), simulations, etc.).	Results, Supplementary Table 3
		c) Report any assessment of direction of causality (e.g., bidirectional MR).	NA
		d) When relevant, report and compare with estimates from non-MR analyses.	Discussion
		e) Consider any additional plots to visualize results (e.g., leave-one-out analyse	NA
14	DISCUSSION: Key Results	Summarize key results with reference to study objectives.	Discussion
15	DISCUSSION: Limitations	Discuss limitations of the study, taking into account the validity of the MR assumptions, other sources of potential bias, and imprecision. Discuss both direction and magnitude of any potential bias, and any efforts to address them.	Discussion
16	DISCUSSION: Interpretation	a) Give a cautious overall interpretation of results considering objectives and limitations. Compare with results from other relevant studies.	Discussion + Conclusion
		b) Discuss underlying biological mechanisms that could be modelled by using the genetic variants to assess the relationship between the exposure and the outcome.	Discussion
		c) Discuss whether the results have clinical or policy relevance, and whether interventions could have the same size effect.	Discussion + Conclusion
17	DISCUSSION: Generalizability	Discuss the generalizability of the study results (a) to other populations (i.e. external validity), (b) across other exposure periods/timings, and (c) across other levels of exposure.	Discussion
18	OTHER INFO: Funding	Give the source of funding and the role of the funders for the present study and, if applicable, for the original study or studies on which the present article	Disclosures and Acknowledgements
19	OTHER INFO: Data + Data Sharing	Present data used to perform all analyses or report where and how the data can be accessed. State whether statistical code is publicly accessible and if so,	Supplementary Table 4
20	OTHER INFO: Conflicts of Interest	All authors should declare all potential conflicts of interest.	Disclosures and Acknowledgements
